# The Enhancement of H_2_ Evolution over Sr_1−1.5x_Tb_x_WO_4_ Solid Solution under Ultraviolet Light Irradiation

**DOI:** 10.3390/ma12091487

**Published:** 2019-05-08

**Authors:** Jia Yang, Xiaorui Sun, Ting Zeng, Yilan Hu, Jianwei Shi

**Affiliations:** Chongqing Key Laboratory of Inorganic Special Functional Materials, College of Chemistry and Chemical Engineering, Yangtze Normal University, Fuling, Chongqing 408100, China; zengting@163.com (T.Z.); huyilan@163.com (Y.H.); shijianwei@163.com (J.S.)

**Keywords:** photocatalysis, semiconductor, solid solution, H_2_ evolution, cocatalyst

## Abstract

In this work, Sr_1−1.5x_Tb_x_WO_4_ (0 ≤ x ≤ 0.2) solid solutions were synthesized via a traditional high-temperature solid state method. Le Bail fitting on the powder *X*-ray diffraction (XRD) pattern showed that these solid solutions are pure phase. Scanning electron microscopy showed that the SrWO_4_ and Sr_0.82_Tb_0.12_WO_4_ samples are composed of micrometer particles and submicron crystallites, respectively. Ultraviolet–visible diffuse reflectance spectra suggested that the bandgaps of Sr_1−1.5*x*_Tb*_x_*WO_4_ are narrower than the undoped sample. The Sr_0.82_Tb_0.12_WO_4_ sample, with the assistance of 1.5 wt % Ru-cocatalyst, exhibits the best performance for H_2_ evolution in 5 vol % aqueous triethanolamine (TEOA), which results in about 6.1 and 2.8 times efficiency improvement compared with the intrinsic SrWO_4_ in methanol and aqueous TEOA, respectively. All the photocatalysts recycled after the photocatalytic reactions showed no degradation when checked by powder XRD.

## 1. Introduction

Photocatalytic technology is a green way to obtain clean energy hydrogen via semiconductor from water under sunlight irradiation [1]. Over the past few decades, various semiconducting materials, including oxide [2], sulfide [3], oxynitride [4], and carbon-based material [5], have been studied extensively in photocatalytic H_2_ evolution. The sulfide and oxynitride materials generally have excellent photocatalytic activities, however, both of them are not stable enough in aqueous solution under light irradiation. For instance, the Zn_1−2y_Ga_1.7_In_0.3_S_4_ and Pt–PdS/CdS samples have outstanding photocatalytic H_2_ evolution activities in aqueous S^2−^/SO_3_^2−^ solutions [6,7]. GaN/ZnO and LaMg_x_Ta_1−x_O_1+3x_N_2−3x_ solid solutions require a cocatalyst or coating material for photocatalytic H_2_ evolution [8,9]. On account of their distinguished electrical conductivity, carbon materials have been utilized to fabricate carbon-based composites [10,11,12]. The majority of oxides were usually only sensitized by UV light, however, if suitable crystal structure can indicate good photocatalytic application [13,14].

The photocatalytic performance of SrWO_4_ has been studied extensively in the field of environmental photocatalysis. Suitable SrWO_4_ crystal structures are able to degrade various dyes and organic materials under ultraviolet light [15,16,17,18,19,20,21]. For example, rhodamine B and 6G were photodegraded by star-like SrWO_4_ microcrystals in water [17]. The methyl orange degradation by SrWO_4_ nanoparticles was about 73% after 60 min ultraviolet light illumination [20]. Ibuprofen was photoelectrooxidized by rice-like SrWO_4_ nanocrystals in Na_2_SO_4_ solution [21]. In addition, there is a single report of H_2_ evolution by SrWO_4_ micro/nanostructures in aqueous methanol under ultraviolet light irradiation [22]. However, the enhancement of H_2_ evolution over SrWO_4_ is lacking.

Generally, fabricating a solid solution is a powerful way to improve photocatalytic performance. For instance, the In_1−*x*_Ni*_x_*TaO_4_ [2] and CuFe_1−*y*_Cr*_y_*S_2_ [23] solid solutions were obtained as optimal samples for photocatalytic applications, where *x* = 0.1 and *y* = 0.4 in each solid solution. The enhancement of photocatalytic activity was due to the narrowing of the bandgap and the changing of the conduction band potential by the doped element [6]. Usually, the bandgap value and conduction band potential of photocatalyst are 1.23 eV and less than 0 V, respectively. In this paper, the photocatalytic H_2_ evolution of scheelite structure SrWO_4_ was improved by doping rare earth ions and cocatalysts. The Sr_1−1.5*x*_Tb*_x_*WO_4_ solid solutions were studied for the first time in aqueous TEOA under ultraviolet light irradiation, and their photocatalytic activity was improved by loading various cocatalysts, such as Cu, Ag, Au, Pt, Ni, and Ru.

## 2. Materials and Methods

### 2.1. Preparations of the Catalysts

The solid state method was applied to synthesize Sr_1−1.5*x*_Tb*_x_*WO_4_ (0 ≤ *x* ≤ 0.2) bulk samples. The starting materials, SrCO_3_ (99.9%, Sinopharm Chemical Reagent Co., Ltd.), Tb_4_O_7_ (99.99%, Alfa Aesar), and WO_3_ (99.5%, Sinopharm Chemical Reagent Co., Ltd.), were used after a pre-calcination at 800 °C to remove possible absorbed moisture or CO_2_. Typically, for their synthesis, Sr_0.82_Tb_0.12_WO_4_, SrCO_3_ (2.62 mmol, 0.3870 g), Tb_4_O_7_ (0.10 mmol, 0.0717 g), and WO_3_ (3.20 mmol, 0.7412 g) were homogenized using an agate mortar, followed by preheating at 800 °C for 10 h. The resultant powder was re-ground thoroughly by hand. Finally, it was heated at 1000 °C for another 15 h in air.

### 2.2. Characterizations

Powder XRD data were collected on a PANalytical X’pert diffractometer equipped with a PIXcel 1D detector (Cu Kα radiation, 1.5406 Å). The operation voltage and current were 40 kV and 40 mA, respectively. Le Bail refinements were performed to obtain cell parameters using the TOPAS software package [24]. Scanning electron microscopy (SEM) was performed on an S4800 at an accelerating voltage of 10 kV. The elemental analysis was performed using SEM fitted with an INCAx-act energy dispersive spectrometer (EDS). Ultraviolet–visible diffuse reflectance spectrum (DRS) was recorded using a Shimadzu UV-3600 spectrometer equipped with an integrating sphere attachment. The analysis range was from 200 to 1200 nm, and BaSO_4_ was used as the reflectance standard. Photoluminescence (PL) spectra were measured on a Hitachi F4600 fluorescence spectrometer at room temperature. The analysis range was from 450 to 600 nm, the excited wavelength was 250 nm, and the PL of the baseline was collected by using an empty glass cuvette.

### 2.3. Theoretical Calculations

Theoretical study on SrWO_4_ was investigated via the Vienna Ab initio Simulation Package (VASP) [25]. The projector augmented-wave (PAW) method implemented in the VASP code was utilized to describe the interaction between the ionic cores and the valence electrons [26]. The generalized gradient approximation (GGA) parameterized by Perdew, Burke, and Ernzerhof (PBE) was employed to describe the exchange-correlation potential in standard density functional theory calculations [27]. For single point energy and density of states, a cutoff energy of 500 eV for the plane-wave basis and the 3 × 3 × 3 Monkhorst–Pack G-centered *k*-point meshes were employed.

### 2.4. Photocatalytic Performance Evaluation

Photocatalytic performances were tested on a gas-closed circulation system equipped with a vacuum device (LabSolar-IIIAG system, Perfect Light Ltd. Co.), a 150 mL Pyrex glass reactor, and a gas sampling port that was directly connected to a gas chromatograph (Shanghai Techcomp-GC7900, TCD detector, molecular sieve 5A, N_2_ gas carrier). In a typical run, 50 mg of catalyst was dispersed by a magnetic stirrer in 50 mL of 5 vol % TEOA aqueous solution. The solution was stirred, and a 10 °C cycling water bath was applied to keep the reaction vessel at a constant temperature. The light irradiation source was generated by an external 500W Hg lamp (CEL-M500, Beijing Au Light Ltd. Co.).

### 2.5. Preparation of the Cocatalysts

The loading of metal cocatalyst on photocatalyst was performed by a photodeposition method using the above setup [14]. For instance, 50 mg Sr_0.8__2_Tb_0.12_WO_4_ sample, together with 1.0 mL of RuCl_3_ (0.97 mg/mL), were mixed in 10 mL of distilled water. This solution was placed in a 150 mL Pyrex glass reactor with an ultrasonic treatment for 10 min, and then the mixture, in the presence of 5 vol % of TEOA, was irradiated using a 500 W high-pressure Hg lamp for 2 h. Finally, the powder sample was collected and washed with deionized water.

## 3. Results and Discussion

**Figure 1:**
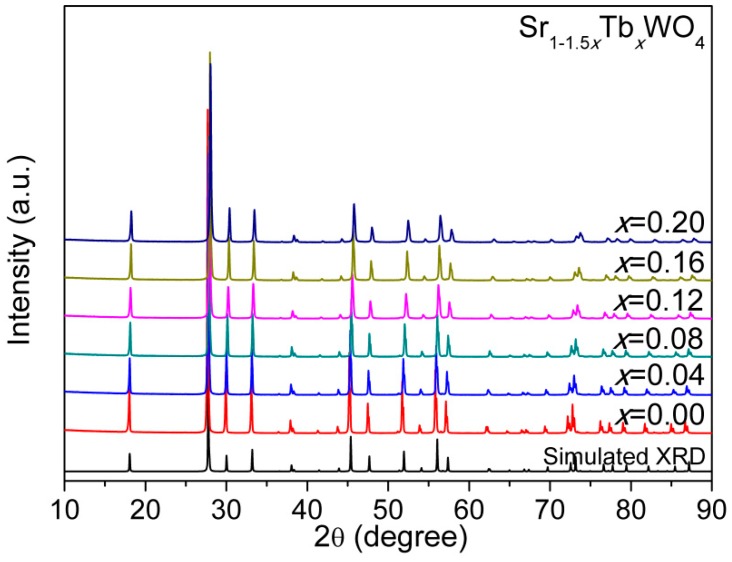
The XRD patterns for the Sr_1−1.5x_Tb_x_WO_4_ (0 ≤ x ≤ 0.20), where the simulated XRD pattern for undoped SrWO_4_ is also given.

**Figure 2:**
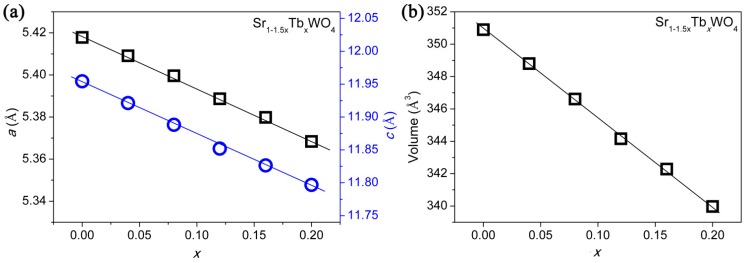
Estimated cell parameters for the Sr_1−1.5x_Tb_x_WO_4_ (0 ≤ x ≤ 0.20) from Le Bail refinements on whole XRD patterns: (**a**) *a* and *c*, (**b**) volume.

Figure 1 presents the whole XRD pattern of SrWO_4_ with substantial doping of Tb^3+^, and the sharp peaks point to the high crystallinity of the as-prepared samples. For Sr_1−1.5*x*_Tb*_x_*WO_4_ (0.00 ≤ *x* ≤ 0.20), it is evident that a pure phase of solid solutions was synthesized without any impurity peaks, when compared to the simulated XRD (ICSD-155793) of SrWO_4_. The peak shift by Tb^3+^-doping is not obvious, but we can determine the change of the cell lattice parameters (*a*, *c*, and *V*) by Le Bail fitting of the whole powder XRD pattern [2,14]. The plot of *a*, *c*, and *V*, along with the increase in *x*, suggests a linear shrinkage (see Figure 2). These results evidently confirm that Tb^3+^ has been successfully incorporated into SrWO_4_ without any structural change.

Scherrer’s formula works best for the nanomaterials (1–100 nm), and the average crystallite sizes of SrWO_4_ and Sr_0.82_Tb_0.12_WO_4_ particles were estimated as per the following equation:

*t* = *Kλ*/*Bcosθ_B_*, (1)

where *t* is the crystallite size of the particle (assuming particles are spherical), *K* = 0.9, *λ* is the wavelength of X-ray radiation, *B* is the full width at half-maximum of the diffracted peak, and *θ_B_* is the diffraction angle [28]. The estimated crystallite sizes based on the (011) peak for SrWO_4_ and Sr_0.82_Tb_0.12_WO_4_ are approximately 89.4 and 50.3 nm, respectively.

**Figure 3:**
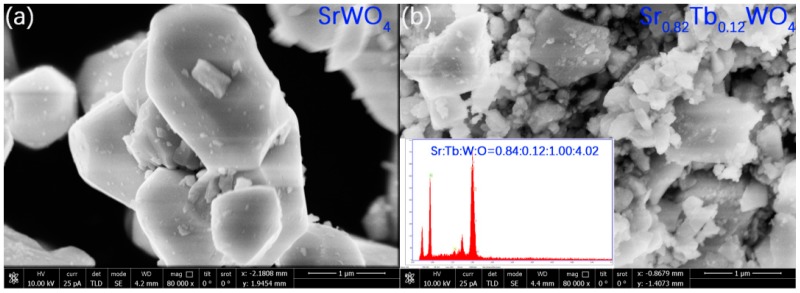
(**a**) SEM image for SrWO_4_. (**b**) SEM and EDS image for Sr_0.82_Tb_0.12_WO_4_.

The as-prepared solid solutions were observed by electron microscopy. Figure 3 shows the SEM images of nondoped SrWO_4_ and Sr_0.82_Tb_0.12_WO_4_ powders prepared under identical conditions. Both particles were well-crystallized, which is consistent with the XRD analysis. The particle size of Sr_0.82_Tb_0.12_WO_4_ powder, 0.1–1 μm, was remarkably smaller than that of nondoped SrWO_4_ powder, 1–2.4 μm. Since the SrWO_4_ and Sr_0.82_Tb_0.12_WO_4_ powders are bulk samples, the particle size determined by SEM is different from the particle size calculated by Scherrer’s formula from XRD, but the relative size of the SrWO_4_ and Sr_0.82_Tb_0.12_WO_4_ powders were not changed. A sharp surface edge was observed for the La-doped SrWO_4_ powder, whereas the surface of nondoped SrWO_4_ was flat. The insert in Figure 3b shows an elemental analysis performed on the Sr_0.82_Tb_0.12_WO_4_ sample, which gave an average atomic ratio of Sr/Tb/W/O = 0.84:0.12:1.00:4.02.

**Figure 4:**
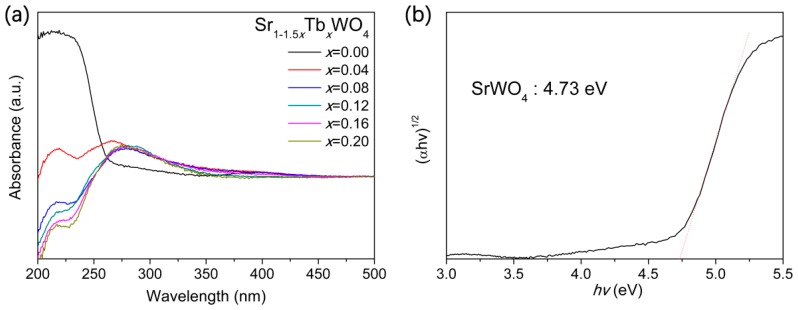
(**a**) UV–vis diffuse reflectance spectrum for Sr_1−1.5*x*_Tb*_x_*WO_4_ solid solutions. (**b**) Estimated bandgap energy *E_g_* with plot of (*αhν*)^2^ against photon energy (*hν*) for SrWO_4_.

Figure 4a shows that the absorption band of SrWO_4_ lies mainly in the UV region, and there is a steep edge which indicates that the absorption band is not due to the transition from impurity energy levels but from the bandgap transition [29]. For most semiconductors, the dependence of the absorption coefficient α on the bandgap energy *E_g_* can be expressed by the following equation: αh*v* = A(*hv* − E*_g_*)^*n*/2^, where *h*, *v*, and A are the Planck constant, light frequency, and proportionality, respectively, and *n* is determined on the basis of the transition type (i.e., *n* = 1 for direct transition, *n* = 4 for indirect transition) [23]. The best fit of (α*hv*) ^2^ vs. *E_g_* was obtained only when *n* is 1, suggesting that direct transition across the energy bandgap of SrWO_4_ is allowed (see Figure 4b). The extrapolated value of *hv* at α = 0 gives an absorption edge energy corresponding to *E_g_*, which is 4.73 eV for SrWO_4_. Note that the reported value of the bandgap is in the range of 3.2–4.96 eV in the literature [16,18,19,20,21], where the difference comes from the different morphology of these samples.

**Figure 5:**
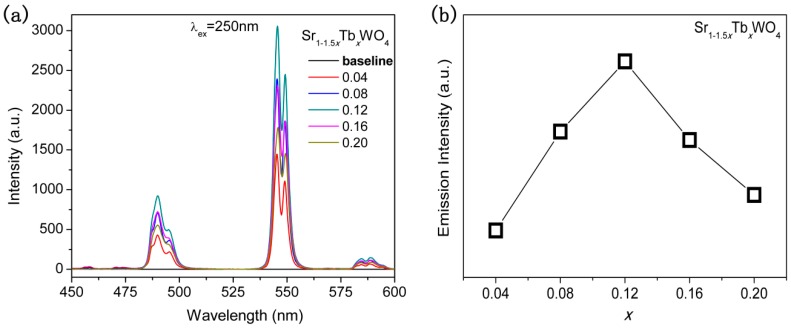
(**a**) The photoluminescence emission spectra of Sr_1−1.5*x*_Tb*_x_*WO_4_ solid solutions by monitoring at 250 nm. (**b**) The emission intensity of the solid solutions against *x*.

As a result of the fluorescence phenomenon of Sr_1−1.5*x*_Tb*_x_*WO_4_ solid solutions, the DRS patterns were interrupted at a range of 200~270 nm (see Figure 4a), in agreement with the excitation wavelength of excitation spectra patterns (see Figure 5a). However, we can conclude that the bandgaps of the Tb-doped samples are smaller than the nondoped SrWO_4_, because the light absorbance of the Tb-doped samples are obviously bigger than the nondoped samples at the wavelengths of 275~400 nm. Sometimes, the weak absorbance of photocatalyst could lead to a significant change of photocatalytic activity, such as Ni-doped InTaO_4_ [2], and carbon dot-decorated C_3_N_4_ [30]. Nevertheless, in our work, the doped sample did not respond to simulated sunlight with 5 vol % TEOA solution. Figure 5a shows the photoluminescence emission spectra of the solid solutions by monitoring the Tb^3+^ emission at 250 nm. The change trends of emission intensity appeared as “volcano” types by increasing the Tb^3+^ content (see Figure 5b). The band–band PL phenomenon was monitored with the light energy approximately equal to the bandgap energy of the sample [31]. Usually, the recombination of photocarriers and PL intensity are positively correlated in photocatalytic studies [32,33], and the photocatalytic activity related to the recombination of photogenerated electron and hole [34]. In other words, the weak PL intensity means that photoexcitation is difficult. The optimal sample regarding photoluminescence is Sr_0.82_Tb_0.12_WO_4_, which means that the photogenerated electrons are easily excited from valence band to conduction band.

**Figure 6:**
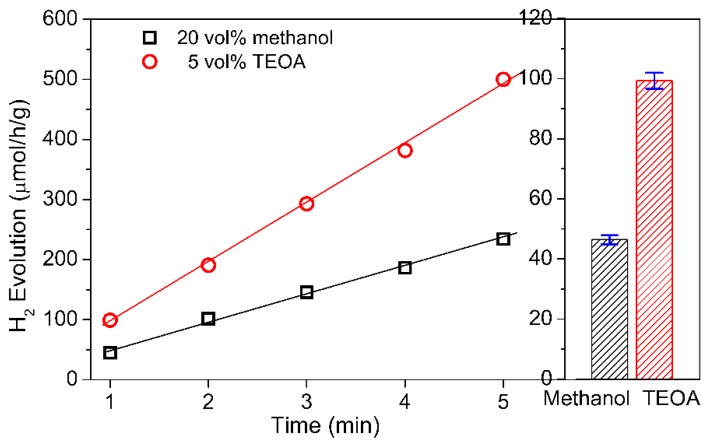
The H_2_ evolution of SrWO_4_ with different electron-donating sacrificial agents. Photocatalytic conditions: 50 mg SrWO_4_ sample, 50 mL solution, 500 W Hg lamp.

To study the photocatalytic activity of SrWO_4_, the aqueous methanol and TEOA solutions were utilized as electron-donating sacrificial agents for H_2_ evolution in our photocatalytic reactions. The H_2_ evolution rate of SrWO_4_ in 5 vol % aqueous TEOA is 2.1 times higher than it is in the 20 vol % aqueous methanol (see Figure 6). Furthermore, compared with the literature [22], the H_2_ evolution rate of SrWO_4_ is almost the same value as in aqueous methanol under UV light.

**Figure 7:**
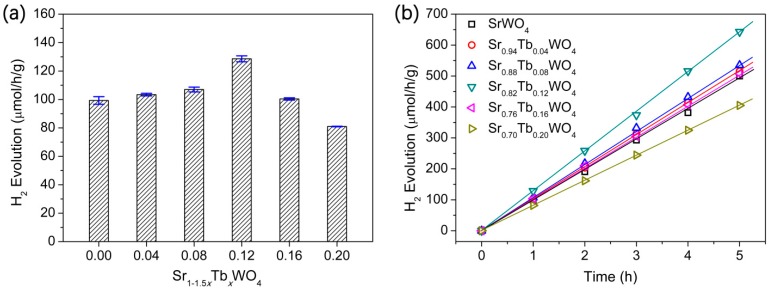
(**a**) The H_2_ evolution rates of Sr_1−1.5*x*_Tb*_x_*WO_4_ samples against increasing *x*. (**b**) The H_2_ evolution of Sr_1−1.5*x*_Tb*_x_*WO_4_ samples against time. Photocatalytic conditions: 50 mg photocatalyst, 50 mL 5% aqueous triethanolamine (TEOA) solution, 500 W Hg lamp.

The photocatalytic activity of the Sr_1−1.5*x*_Tb*_x_*WO_4_ solid solutions toward the H_2_ production at 5 h reaction times, in the presence of 5 vol % TEOA as electron-donating sacrificial agent under UV light, is shown in Figure 7. The H_2_ production rates were changed by increasing the Tb^3+^ content in a trend of “volcano” type, which is in agreement with the photoluminescence emission intensity. Figure 7a shows that the optimum sample is Sr_0.82_Tb_0.12_WO_4_, and that its H_2_ evolution rate is 128.6 μmol/h/g. Compared with the undoped SrWO_4_, the photocatalytic activity of Sr_0.82_Tb_0.12_WO_4_ is improved about 1.3 times, which can be explained as follows: (1) The light absorption of the solid solutions are enhanced via the increasing of Tb^3+^, leading to the improvement of photocatalytic activity [35]; (2) The non-equivalent doping of Tb^3+^ to replace Sr^2+^ in SrWO_4_ can fabricate a metal vacancy in the bulk and surface of the material, which may constitute a recombination center [36]. Figure 7b shows that the H_2_ evolution of the solid solutions is linear against time.

**Figure 8:**
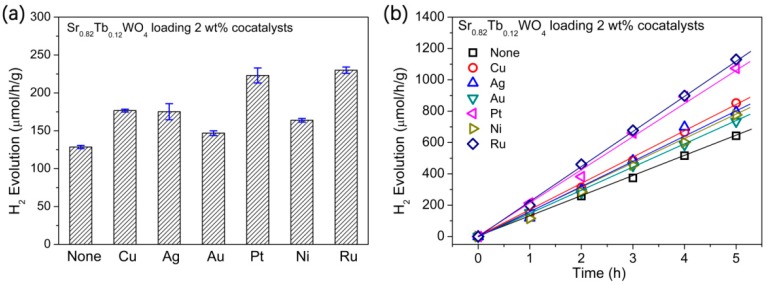
(**a**) The H_2_ evolution rates of Sr_0.82_Tb_0.12_WO_4_ samples with different cocatalysts. (**b**) The H_2_ evolution of Sr_0.82_Tb_0.12_WO_4_ samples against time. Photocatalytic conditions: 50 mg photocatalyst, 50 mL 5% TEOA solution, 500 W UV light.

The H_2_ evolution rates of SrWO_4_ micro/nanostructures were obviously improved by Pt- and Ru-cocatalysts [22]. Usually, metal cocatalysts offer photocatalytic water reduction sites, which means that photogenerated electrons prefer to move to metal cocatalysts [37,38]. In our experiment, the various cocatalysts, including Cu, Ag, Au, Pt, Ni, and Ru, were loaded onto the Sr_0.82_Tb_0.12_WO_4_ sample to promote photocatalytic activity (Figure 8a). The Ru-cocatalyst loaded onto the photocatalysts had the optimum H_2_ production rate (128.6 μmol/h/g), which is 1.3 times that of the nonloaded SrWO_4_. Figure 8b shows that the H_2_ evolution of the cocatalyst-loaded Sr_0.82_Tb_0.12_WO_4_ samples is linear against time.

**Figure 9:**
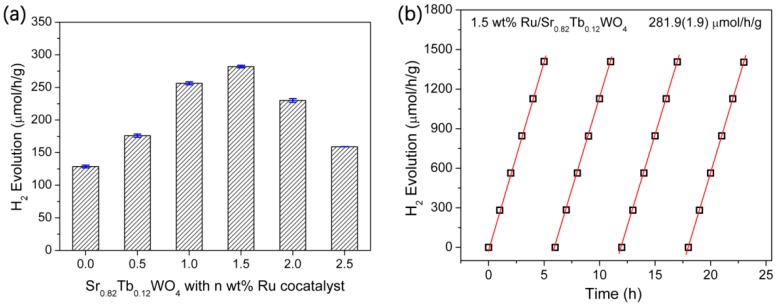
(**a**) The H_2_ evolution rates of Sr_0.84_Tb_0.12_WO_4_ solid solutions with differing amounts of cocatalyst. (**b**) A long-term photocatalytic reaction over Sr_0.82_La_0.12_WO_4_ loaded with 1.5 wt % Ru-cocatalyst. Photocatalytic conditions: 50 g photocatalyst, 50 mL 5% TEOA solution, 500 W Hg lamp.

**Figure 10:**
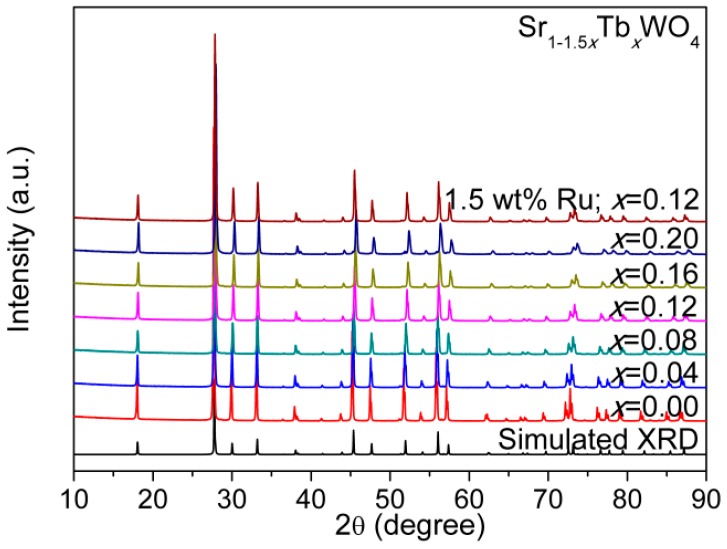
The XRD patterns of the solid solutions, and the recycled samples after photocatalysis.

The optimum usage of Ru-cocatalyst for the Sr_0.82_Tb_0.12_WO_4_ sample was observed to be 1.5 wt % (Figure 9a). Its H_2_ evolution rate was 281.9 μmol/h/g, which is 2.8 times that of the nonloaded SrWO_4_. The photocatalytic activity of the optimum sample is stable after 30 h UV light irradiation, as shown in Figure 9b. In our work, the control experiments were performed without any sacrificial donor. However, the SrWO_4_, Sr_0.82_Tb_0.12_, and 1.5 wt % Ru/Sr_0.82_Tb_0.12_WO_4_ samples had no detectable photocatalytic activity in H_2_ evolution. The XRD patterns of the as-prepared photocatalysts showed no obvious change before and after photocatalytic reaction (see Figure 10).

In order to determine that the enhancement is really due to Tb or Ru for the optimum 1.5 wt % Ru-loaded Sr_0.82_Tb_0.12_WO_4_ sample, we compared the photocatalytic H_2_ evolution of SrWO_4_, 1.5 wt % Ru-loaded SrWO_4_, Sr_0.82_Tb_0.12_WO_4_, and 1.5 wt % Ru-loaded Sr_0.82_Tb_0.12_WO_4_ (see Table 1). Indeed, the value of [3]:[1] is larger than the value of [2]:[1], which means the doped Tb is better than the loaded Ru for H_2_ evolution. The value of [4]:[2] is larger than [4]:[3], which indicates that the doped Tb is also better. Therefore, we can conclude that Tb is relatively more important for the H_2_ evolution of SrWO_4_ in our experiment.

**Figure 11:**
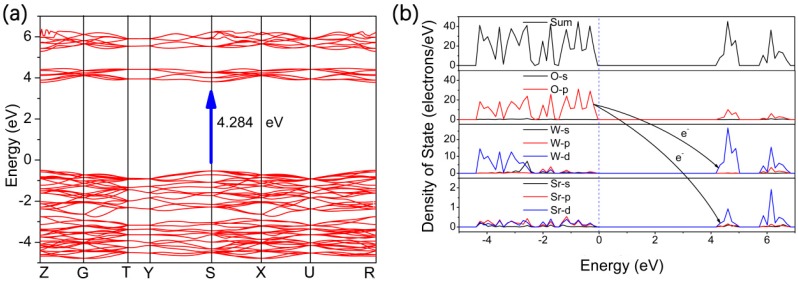
Theoretical calculations band structure and density of states for SrWO_4_.

To obtain further insight into the photocatalytic activity of SrWO_4_, the electronic structure of SrWO_4_ was investigated by VASP calculations. Figure 11 shows the energy band dispersion and density of states (DOS). Although the bandgap (4.284 eV) from VASP calculations is usually underestimated, it nonetheless often provides important insight into the physicochemical behavior of the investigated materials [6,14]. The material is a direct bandgap semiconductor, as has been revealed from the DRS pattern. As shown in Figure 11a, both the valence band maximum and the conduction band minimum of SrWO_4_ are located at the S point of the Brillouin zone, which explains the features of the measured DRS pattern, as does the reported literature [22]. This is in agreement with the inference from the DRS pattern. Both the lowly dispersive valence bands and the conduction bands should not be beneficial for the transport of the photoexcited electrons and holes. Their lowly dispersive bands, in turn, are likely to result in high recombination of electron hole pairs and, thus, account for the low photocatalytic activity of SrWO_4_. The total DOS shows that the bands of SrWO_4_ can be classified into two parts (see Figure 11b). The top of the valence band is completely dominated by the O 2p orbital, while the bottom of the conduction band is mainly constituted by the W 5d orbital and the Sr 4d orbital. In addition, the band structure indicates that charge transfer upon photoexcitation occurs from the O 2p orbital to the empty W 5d orbital and Sr 4d orbital.

## 4. Conclusions

We prepared Sr_1−1.5*x*_Tb*_x_*WO_4_ solid solutions by high-temperature solid state reaction. Le Bail fitting on powder XRD verified high purity and crystallinity of the as-prepared samples, and the SEM images showed that the SrWO_4_ and Sr_0.82_La_0.12_WO_4_ were composed of micron and submicron crystallites, respectively. DRS and theoretical calculations of SrWO_4_ samples suggested a wide bandgap characteristic, which was 4.73 eV, assuming the direct semiconductor model. The valence band maximum was composed of O 2p orbitals, and the conduction band minimum was composed of both W 5d and Sr 4d orbitals. The H_2_ evolution rates of SrWO_4_ samples were 46.4 μmol/h/g and 99.3 μmol/h/g, with the 20 vol % methanol and 5 vol % TEOA aqueous solutions, respectively. The photocatalytic activity of SrWO_4_ was improved by doping Tb^3+^ and then loading cocatalysts. The 1.5 wt % Ru-cocatalyst loaded onto Sr_0.82_Tb_0.12_WO_4_ improved the H_2_ evolution rate to 281.7 μmol/h/g. This photocatalyst remained stable and active even after five cycles (25 h in total). The improvement of photocatalytic performance for 1.5 wt % Ru/Sr_0.82_Tb_0.12_WO_4_ was mainly due to the doped Tb in the crystal structure. These samples did not respond to simulated sunlight with the 5 vol % TEOA, and had no detectable photocatalytic activity in water under UV light. Our study demonstrates the photocatalytic H_2_ evolution of Sr_1−1.5*x*_Tb*_x_*WO_4_, and our preliminary attempt of Tb^3+^-doping and loading of various cocatalysts did, indeed, improve the activity. The next steps involve extending the studies to other rare earth ions to produce materials that absorb more of the visible region of the spectrum, or to complete the water-splitting reaction.

## Figures and Tables

**Table 1:** The photocatalytic H_2_ evolution of four samples in 5 vol % TEOA under UV light.

Photocatalyst	[1] SrWO_4_	[2] 1.5 wt % Ru-Loaded SrWO_4_	[3] Sr_0.82_Tb_0.12_WO_4_	[4] 1.5 wt % Ru-Loaded Sr_0.82_Tb_0.12_WO_4_
**H_2_ evolution (μmol/h/g)**	99.3	117.4	128.6	281.9
**ratio**		[2]:[1] = 1.18	[3]:[1] = 1.30	[4]:[1] = 2.84; [4]:[2] = 2.40; [4]:[3] = 2.19
